# Cationic lipid-nanoceria hybrids, a novel nonviral vector-mediated gene delivery into mammalian cells: investigation of the cellular uptake mechanism

**DOI:** 10.1038/srep29197

**Published:** 2016-07-06

**Authors:** Joydeep Das, Jae Woong Han, Yun-Jung Choi, Hyuk Song, Ssang-Goo Cho, Chankyu Park, Han Geuk Seo, Jin-Hoi Kim

**Affiliations:** 1 Department of Stem Cell and Regenerative Biology, Humanized Pig Research Center (SRC), Konkuk University, Seoul 143-701, South Korea

## Abstract

Gene therapy is a promising technique for the treatment of various diseases. The development of minimally toxic and highly efficient non-viral gene delivery vectors is the most challenging undertaking in the field of gene therapy. Here, we developed dimethyldioctadecylammonium bromide (DODAB)–nanoceria (CeO_2_) hybrids as a new class of non-viral gene delivery vectors. These DODAB-modified CeO_2_ nanoparticles (CeO_2_/DODAB) could effectively compact the pDNA, allowing for highly efficient gene transfection into the selected cell lines. The CeO_2_/DODAB nanovectors were also found to be non-toxic and did not induce ROS formation as well as any stress responsive and pro-survival signaling pathways. The overall vector performance of CeO_2_/DODAB nanohybrids was comparable with lipofectamine and DOTAP, and higher than calcium phosphate and DEAE-dextran for transfecting small plasmids. The increased cellular uptake of the nanovector/DNA complexes through clathrin- and caveolae-mediated endocytosis and subsequent release from the endosomes further support the increased gene transfection efficiency of the CeO_2_/DODAB vectors. Besides, CeO_2_/DODAB nanovectors could transfect genes *in vivo* without any sign of toxicity. Taken together, this new nano-vector has the potential to be used for gene delivery in biomedical applications.

Gene therapy has been recognized as a promising technique to treat genetic disorders and cancers. Gene therapy involves the reparation of defective genes or the incorporation of new functional genes into the cells^1^,^2^. However, the negatively charged genes cannot traverse the negatively charged cell membrane effectively without the assistance of gene delivery vectors. Gene delivery vectors include both viral^3^,^4^,^5^ and non-viral systems^6^,^7^,^8^. Although viral vectors show high transaction efficiency, they are limited in terms of DNA packaging and are also hazardous to humans^9^. Therefore, non-viral vectors have gained prominence because they are largely biocompatible, easily functionalized and varied structurally, and have the potential to carry diverse genetic materials into living cells^10^. Non-viral gene delivery vectors have to overcome three important barriers during gene delivery: (1) DNA entry across the cell membrane, (2) protection of DNA bound to the vectors and subsequent release of DNA, and (3) DNA entry into the nucleus. Therefore, development of minimally toxic and highly efficient non-viral gene delivery vectors is the most challenging undertaking in the field of gene therapy^11^.

In recent years, inorganic nanoparticle-based gene delivery vectors have gained the attention of researchers due to their unique physical and chemical properties^12^. Several types of inorganic nanoparticles can form stable complexes with DNA and deliver it into living cells. These include silica nanoparticles^13^,^14^, quantum dots^15^, Au nanoparticles^16^,^17^,^18^, carbon nanotubes^19^,^20^, hybrid nanoparticles^21^, etc. Herein, for the first time, we introduce nanoceria (CeO_2_) as a non-viral gene delivery vector. Nanoceria is well known for its excellent antioxidant activity^22^,^23^,^24^,^25^,^26^,^27^. Nanoceria is a reported mimic for superoxide dismutase (SOD) with catalytic efficiency surpassing that of SOD itself^28^,^29^. Nanoceria has the ability to alter its valence state (between Ce^3+^ and Ce^4+^) and create oxygen defects on its surface. The catalytic activity of nanoceria is derived directly from this property^30^,^31^. Therefore, it is quite reasonable to use nanoceria as a gene delivery vector due to its biocompatible nature. Liu *et al*.^32^ demonstrated that instead of having a negative surface zeta potential at physiological pH, nanoceria exhibits strong DNA binding ability. However, they also showed that nanoceria could not prevent the migration of DNA upon gel electrophoresis, possibly due to the resultant negative charge of the complex. For effective gene delivery, the charge of the nanoparticle-DNA complex should be positive to facilitate intracellular uptake. Therefore, the nanoparticle should possess sufficient positive charge to confer a resultant positive charge to the nanoparticle-DNA complex. In the present study, we utilize dimethyldioctadecylammonium bromide (DODAB), a commercially available cationic lipid, for the surface modification of nanoceria. The interaction of DODAB with DNA and its application as a gene delivery vector has been evaluated in several prior studies^33^,^34^,^35^,^36^,^37^,^38^. However, due to its low transfection efficiency and enhanced cytotoxicity, DODAB could not been commercialized and applied for gene therapy.

Herein, we propose that by combining the advantage of DODAB as a gene delivery vector and the biocompatible nature of nanoceria to prepare a one-particle system (CeO_2_/DODAB), high gene delivery efficiency can be achieved. To test this hypothesis we (i) prepared negatively charged nanoceria from ammonium cerium(IV) nitrate with subsequent characterization, (ii) characterized positively charged nanoceria prepared via surface modification using DODAB, (iii) evaluated the size and surface zeta potentials of the nanoparticles and nanoparticle-DNA complexes, (iv) assessed the DNA complexing ability and protection against DNase I; (v) checked the *in vitro* transfection efficiency and cytocompatibility of the nanoparticles and intracellular distribution of the nanoparticle-DNA complexes, (vi) investigated the intracellular uptake pathways of the nanoparticle-DNA complexes, and (vii) evaluated the *in vivo* transfection efficiency and biocompatibility of the nanoparticles.

## Results

### Preparation and characterization of nanoceria (CeO_2_), DODAB-modified nanoceria (CeO_2_/DODAB), and CeO_2_/DODAB-pDNA complexes

**Figure 1:**
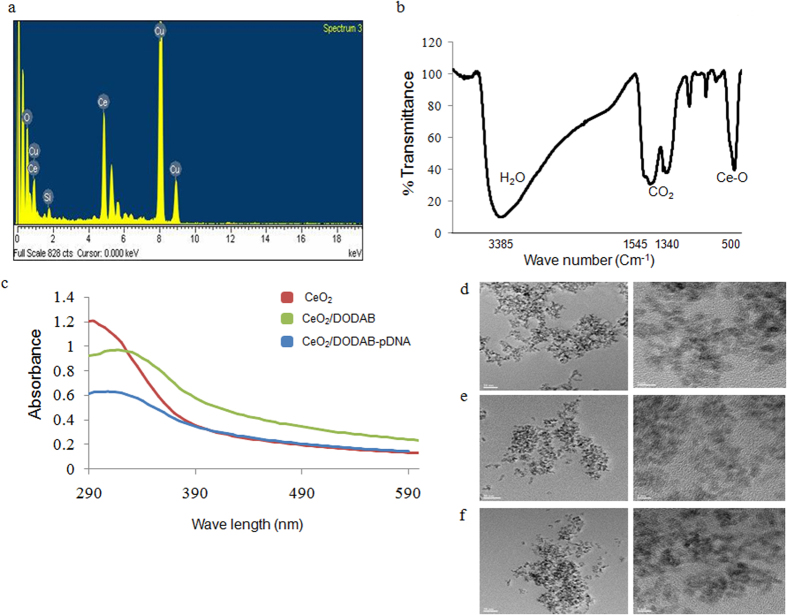
Characterization of nanoparticles and nanoparticle-pDNA complexes. (**a**) EDS spectrum of CeO_2_; (**b**) FTIR spectrum of CeO_2_; (**c**) UV spectra of CeO_2_, CeO_2_/DODAB, and CeO_2_/DODAB–pDNA complexes; (**d**–**f**) TEM images of CeO_2_, CeO_2_/DODAB and CeO_2_/DODAB–pDNA complexes at different magnifications.

In the present study, nanoceria (CeO_2_) was prepared by simply refluxing ammonium cerium(IV) nitrate and urea according to the method of Tsai^39^. The synthesized CeO_2_ was characterized by energy dispersive spectroscopy (EDS) and Fourier transform infrared (FTIR) spectroscopic analyses. The EDS spectrum showed characteristic peaks of Ce and O and was devoid of any impurity peaks (Fig. 1a). However, a Cu peak arising from the TEM grid and a Si peak from the detector were observed. The chemical nature of CeO_2_ was also verified from the FTIR spectrum, which showed a strong absorption band at 500 cm^−1^ due to the Ce-O stretching vibration (Fig. 1b). Infrared absorption bands were also observed at 3385 cm^−1^, 1545 cm^−1^, and 1340 cm^−1^ due to water and CO_2_ molecules adsorbed on the nanoparticle surface (Fig. 1b). After confirming the synthesis of CeO_2_, CeO_2_/DODAB was prepared by simply mixing CeO_2_ and DODAB in a 1:2 mole ratio. The amount of DODAB (6.30%) bound to the nanoceria surface was calculated from the amount of nitrogen present in CeO_2_/DODAB (Supplementary Table 1). The optical absorbance of synthesized CeO_2_ was checked by acquisition of the UV spectrum, which showed a distinct absorption band at 295 nm and was devoid of impurity peaks (Fig. 1c). However, in the case of CeO_2_/DODAB, the absorption band appeared at 315 nm (Fig. 1c). The CeO_2_/DODAB-pDNA complex (CeO_2_/DODAB to pEGFP-N1 mass ratio = 30) showed a broad band spanning 315–320 nm (Fig. 1c). TEM analysis showed almost spherical particles of synthesized CeO_2_ having diameters in the range of 3–4 nm (Fig. 1d). However, the apparent increase in the size of the CeO_2_/DODAB and CeO_2_/DODAB-pDNA complexes was not clear from the TEM images (Fig. 1e,f). The processes of DODAB deposition on the CeO_2_ surface and complex formation between CeO_2_/DODAB and pDNA were also monitored by dynamic light scattering (DLS) and zeta potential analyses. The DLS data suggested that the average diameter of CeO_2_, CeO_2_/DODAB, and CeO_2_/DODAB-pEGFPN1 were 291 ± 7, 370 ± 19, and 461 ± 14 nm respectively (Table 1) in water. Nanoparticles appear larger by DLS compared to by TEM analysis because of the solvation/hydration of nanoparticles. The results can be explained by the fact that in DLS measurement, the mean diameter is calculated from the diffusional properties of dynamic nanoparticles in hydrated state, whereas in TEM analysis, the mean primary particle diameter is calculated in dried state^40^,^41^. The zeta potential values of CeO_2_, CeO_2_/DODAB, and CeO_2_/DODAB-pEGFPN1 were −24 ± 0.9 mV, +41 ± 0.44 mV and +36 ± 0.4 mV respectively (Table 1) in water. The hydrodynamic diameter and zeta potential values of CeO_2_/DODAB-pGL3 (mass ratio = 30) and DODAB-pGL3 (N/P ratio = 8) complexes were also checked; the values were found to be 464 ± 74 nm and +34 ± 0.4 mV respectively for CeO_2_/DODAB-pGL3, and 583 ± 72 nm and +37 ± 0.6 mV respectively for DODAB-pGL3 in water (Table 1).

### pDNA binding affinity of CeO_2_/DODAB and protection of pDNA against nucleases

Herein, we evaluated the binding affinity of the DODAB-modified CeO_2_ nanoparticles (CeO_2_/DODAB) for pEGFP-N1 (100 ng) by agarose gel (1.0% w/v) retardation assay after synthesizing complexes with different mass ratios of CeO_2_/DODAB to pEGFP-N1. Supplementary Figure 1a shows that CeO_2_/DODAB was able to retard pDNA when used at mass ratios of CeO_2_/DODAB to pEGFP-N1 = 10 or above, indicating successful DNA binding via electrostatic interactions. After confirming the effective DNA binding ability of the CeO_2_/DODAB nanoparticles, we evaluated the integrity of pDNA bound to CeO_2_/DODAB against treatment with nucleases. Pristine pDNA and unbound pDNA at mass ratio of CeO_2_/DODAB to pEGFP-N1 = 5 were completely degraded after 30 min of treatment with DNase I, whereas pDNA bound to CeO_2_/DODAB remained stable at mass ratios of CeO_2_/DODAB to pEGFP-N1 = 10 or above (Supplementary Figure 1b). In order to quantitatively evaluate the protection of pDNA against DNase I, the CeO_2_/DODAB-pDNA complex (CeO_2_/DODAB to pEGFP-N1 mass ratio = 30) was treated with DNase I, and the bound DNA was then released by SDS treatment. Supplementary Figure 1c demonstrates that DNA complexed with CeO_2_/DODAB did not undergo significant degradation after DNase I treatment.

### Gene transfection efficiency and cytocompatibility of CeO_2_/DODAB in HEK293 cells

**Figure 2:**
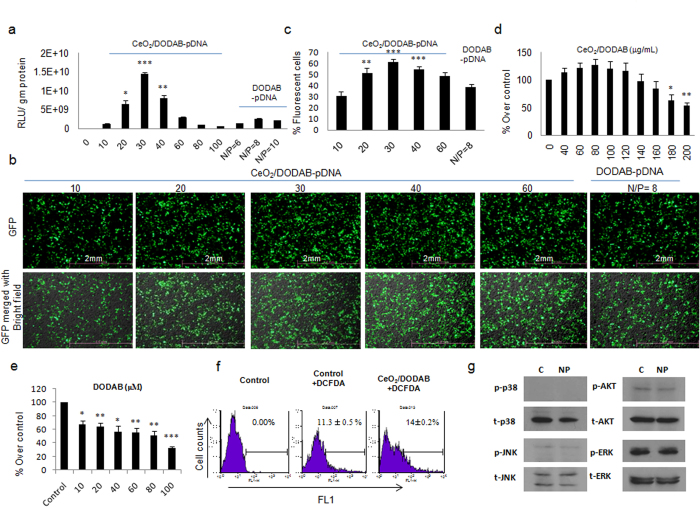
Gene transfection efficiency and cytocompatibility of CeO_2_/DODAB in HEK293 cells. (**a**) Transfection efficiency measured by Luciferase assay; (**b**) fluorescence microscopic images of cells transfected with pEGFP-N1; (**c**) quantification of GFP-positive cells by flow cytometry analysis. The CeO_2_/DODAB-pDNA and DODAB-pDNA complexes were prepared with various mass ratios and N/P ratios, respectively, using 1 μg pDNA. All values are expressed as mean ± SD. *p < 0.05, **p < 0.01, and ***p < 0.001 versus the DODAB-pDNA group; (**d**,**e**) cell viability relative to the control (100%). Cells were treated with different concentrations of CeO_2_/DODAB (40–200 μg/mL) or DODAB (20–100 μM) for 6 hrs and cell viability was measured after 48 hrs using the Cell Counting Kit-8 (CCK-8); (**f**) ROS formation by flow cytometry analysis after 24 hrs of transfection using pDsRed-Monomer-N1vector-CeO_2_/DODAB complexes. All values are expressed as mean ± SD. *p < 0.05, **p < 0.01 and **p < 0.01 versus the non-treated group; (**g**) Western blot analysis of stress responsive and pro-survival pathways after 24 hrs of transfection using pDsRed-Monomer-N1vector-CeO_2_/DODAB complexes.

The gene transfection efficiency of the CeO_2_/DODAB and DODAB alone was first checked by Luc gene expression. We used CeO_2_/DODAB in different mass ratios (10–100) with respect to 1 μg of pGL3-Control (1.5 nano mole bp). Elemental analysis showed that 10 μg of CeO_2_/DODAB contained 1 nano mole DODAB (1 nano mole nitrogen). The N/P ratios for the CeO_2_/DODAB-pDNA complexes corresponding to different mass ratios are presented in Supplementary Table 2. DODAB/pGL3 complexes were also prepared with nitrogen to phosphate ratios of 6 to 10 based on a previous report that DODAB exhibited optimum transfection efficiency at an N/P ratio of 8^42^. Figure 2a shows that the highest transfection efficiency for CeO_2_/DODAB was obtained at the mass ratio of 30 (N/P = 1). On the other hand, the highest transfection efficiency for DODAB was obtained at the N/P ratio of 8, which is consistent with the previous report^42^ (Fig. 2a). The Luc expression reached 1.45 × 10^10 ^RLU/gm protein for CeO_2_/DODAB, while the Luc expression was around 2.62 × 10^9 ^RLU/gm protein using DODAB alone as a vector at their respective optimal ratios. The gene transfection efficiency of the CeO_2_/DODAB nanovectors was further analyzed by EGFP gene expression. Fluorescence microscopy and flow cytometry analyses also demonstrated that the highest transfection efficiency for CeO_2_/DODAB was obtained at the mass ratio of 30 (N/P = 1) (Fig. 2b,c), which is consistent with the Luc gene expression analysis. The transfection efficiency was 61%, where as for DODAB (N/P = 8) alone the transfection efficiency was only 39% (Fig. 2b,c).

The cytotoxicity of the synthesized CeO_2_/DODAB vectors was evaluated within the concentration range of 40–200 μg/mL, which corresponds to mass ratios of 20–100 for gene transfection experiments. The synthesized CeO_2_/DODAB vectors were found to be nontoxic at a concentration upto 140 μg/mL (which is 2.3 times greater than the concentration at which CeO_2_/DODAB shows optimum transfection efficiency) (Fig. 2d). At a concentration of 160 μg/mL (which is 2.7 times greater than the concentration at which CeO_2_/DODAB shows optimum transfection efficiency), almost 84% of the cells were metabolically active (Fig. 2d). In contrast, DODAB was cytotoxic at a concentration of 24 μm (showing optimum transfection efficiency), and when the concentration was increased to 2.5 times the optimum concentration, the cell viability was reduced to around 55% (Fig. 2e). We further checked the reactive oxygen species (ROS) formation by flow cytometry and the effects on stress responsive and pro-survival pathways after 24 hrs of transfection using 4.7 kb pDsRed-Monomer-N1vector-CeO_2_/DODAB complexes. Figure 2f demonstrated that transfection with CeO_2_/DODAB (14% DCF positive population) only increased around 3% ROS compared with the control nontreated cells (11% DCF positive population). Besides, CeO_2_/DODAB also did not induce any stress responsive signaling pathways, such as JNK and p38 as well as pro-survival signaling pathways, such as ERK and AKT (Fig. 2g).

### Gene transfection efficiency and cytotoxicity of CeO_2_/DODAB, Lipofectamine 2000, DOTAP, DEAE-Deaxtran and calcium phosphate in HEK293, MCF-7 and HepG2 cells

**Figure 3:**
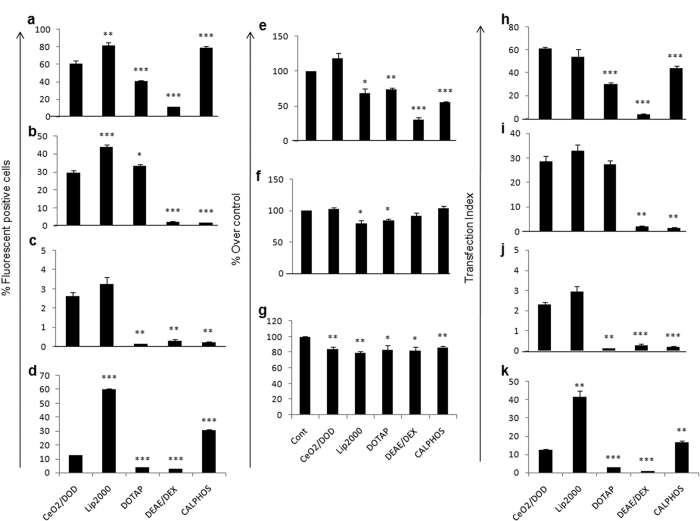
Gene transfection efficiency and cytotoxicity of CeO_2_/DODAB, Lipofectamine 2000, DOTAP, DEAE-Deaxtran and calcium phosphate in HEK293, MCF-7 and HepG2 cells. (**a**–**c**) Quantification of GFP-positive cells by flow cytometry analysis after transfection with pEGFPN1 in HEK293, MCF-7 and HepG2 cells respectively; (**d**) quantification of RFP-positive cells by flow cytometry analysis after transfection with pTRIPZ in HEK293 cells. All values are expressed as mean ± SD. *p < 0.05, **p < 0.01, and ***p < 0.001 versus the CeO_2_/DODAB group; (**e**–**g**) cell viability relative to the control (100%) in HEK293, MCF-7 and HepG2 cells respectively. Cells were treated with different transfection agents at the same concentration that was used for transfection for 6 hrs and cell viability was measured after 48 hrs using the Cell Counting Kit-8 (CCK-8). All values are expressed as mean ± SD. *p < 0.05, **p < 0.01, and **p < 0.01 versus the non-treated group; (**h**–**j**) Transfection Index (the product of percentage transfection and viability) for pEGFP transfection in HEK293, MCF-7 and HepG2 cells respectively. In HEK293 cells, for CeO_2_/DODAB group, the cell viability was taken 100% instead of 120%. (**k**) Transfection Index (the product of percentage transfection and viability) for pTRIPZ transfection in HEK293 cells. All values are expressed as mean ± SD. *p < 0.05, **p < 0.01, and ***p < 0.001 versus the CeO_2_/DODAB group.

Further, we have compared the gene transfection efficiency of CeO_2_/DODAB nanovector with Lipofectamine 2000, DOTAP, DEAE-Dextran and calcium phosphate transfection methods by EGFP gene expression analysis in HEK293, MCF-7 and HepG2 cells. Fluorescence microscopy and flow cytometry analyses demonstrated that the transfection efficiency of Lipofectamine 2000, DOTAP, DEAE-Dextran and calcium phosphate were around 82%, 41%, 11% and 79% respectively in HEK293 cells (Fig. 3a, Supplementary Figure 2a). The transfection efficiency of CeO_2_/DODAB was higher than DOTAP and DEAE-Dextran (Fig. 3a). Although Lipofectamine 2000 and calcium phosphate displayed much higher transfection efficiency compared to CeO_2_/DODAB (Fig. 3a), but reduced the cell viability upto 70% and 55% respectively, where as CeO_2_/DODAB did not induce any cytotoxicity (Fig. 3e). Further, we have checked the Transfection Index (TI) of the individual transfection reagents by calculating the product of transfection efficiency and cell viability. We observed that the TI of CeO_2_/DODAB (60%) was comparable with Lipofectamine 2000 (56%), and significantly higher than the other transfection reagents (Fig. 3h).

Fluorescence microscopy and flow cytometry analyses showed that the transfection efficiency for CeO_2_/DODAB, Lipofectamine 2000, DOTAP, DEAE-Dextran and calcium phosphate were around 30%, 44%, 33%, 2% and 1% respectively in MCF-7 cells (Fig. 3b, Supplementary Figure 3a). Although Lipofectamine 2000 and DOTAP displayed higher transfection efficiency compared to CeO_2_/DODAB (Fig. 3b), but reduced the cell viability upto 80% and 85% respectively, where as CeO_2_/DODAB did not induce any cytotoxicity (Fig. 3f). However, the TI of CeO_2_/DODAB (30%) was comparable with lipofectamine 2000 (35%) and DOTAP (28%), and significantly higher than the other transfection reagents (Fig. 3i). In HepG2 cells, the transaction efficiency of CeO_2_/DODAB and Lipofectamine 2000 was found to be around 3% (Fig. 3c, Supplementary Figure 4a) and all the transfection reagents showed moderate cytotoxic effects (cell viability reduced upto 82–86%) (Fig. 3g).

We have also checked the ability of CeO_2_/DODAB to transfect large plasmid (pTRIPZ, 13.34 kb) and compared the transfection efficiency with other transfection reagents. In HEK293 cells, CeO_2_/DODAB could transfect around 12.63% cells, which was still higher than DOTAP and DEAE-Dextran (<3%), where as the transfection efficiency of calcium phosphate and Lipofectamine 2000 were around 30.4% and 60.4% respectively (Fig. 3d, Supplementary Figure 2b). We observed that the TI of CeO_2_/DODAB (12.63%) was also less than Lipofectamine 2000 (41.53%) and calcium phosphate (16.83%), and significantly higher than the other transfection reagents (Fig. 3k). On the other hand, the transfection ability of all the above mentioned transfection reagents including CeO_2_/DODAB and Lipofectamine 2000 to transfect a 13.34 kb plasmid was very poor (<1%) in both MCF-7 and HepG2 cells (Supplementary Figure 3b, 4b).

### Cellular uptake and distribution of CeO_2_/DODAB nanovector DNA complexes in HEK293 cells

**Figure 4:**
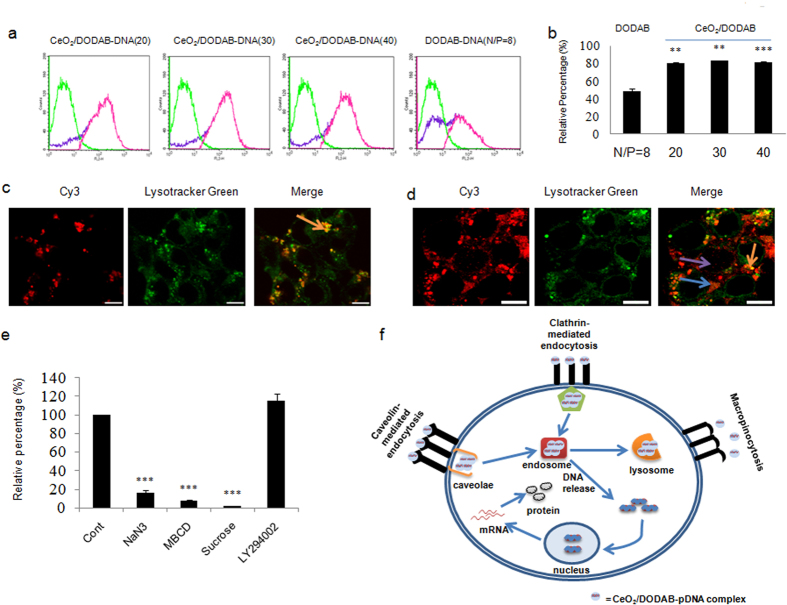
Intracellular uptake and distribution of CeO_2_/DODAB-DNA complexes in HEK293 cells. (**a**,**b**) Flow cytometry analysis of the uptake of CeO_2_/DODAB-Cy3-DNA and DODAB-Cy3-DNA complexes. The CeO_2_/DODAB-Cy3-DNA and DODAB-Cy3-DNA complexes were prepared with various mass ratios and N/P ratio = 8 respectively, using 1 μg Cy3-DNA. Green line represents the population of Cy3 negative cells (background), violet line represents the population of Cy3 positive cells after transfection with nanoparticles or DODAB, pink line (subtraction of background from Cy3 positive population) represents the population of real Cy3 positive cells. All values are expressed as mean ± SD. **p < 0.01 and ***p < 0.001 versus the DODAB-Cy3-DNA group; (**c**,**d**) Confocal microscopic images of intracellular trafficking and localization of CeO_2_/DODAB-Cy3-DNA complexes after 3 hrs and 6 hrs of transfection, respectively. The CeO_2_/DODAB-Cy3-DNA complexes were prepared with a mass ratio of 30 using 1 μg Cy3-DNA. Bright green regions indicate lysosomes and endosomes stained with LysoTracker Green. Red regions indicate Cy3-DNA. Blue arrow indicates cytosolic distribution of Cy3-DNA. Violet arrow indicates nuclear distribution of Cy3-DNA. Orange arrow indicates lysosomal/endosomal distribution of Cy3-DNA. The scale bar is 10 um, (**e**) Gene transfection efficiency was measured by Luciferase assay after transfection with CeO_2_/DODAB-pGL3 (mass ratio = 30 with 1 μg pDNA). The cells were either cultured at 37 °C as a control (Cont) or pretreated with NaN_3,_ MBCD, sucrose, or LY294002. All values are expressed as mean ± SD. ***p < 0.001 versus the control group; (**f**) Schematic diagram of the gene delivery pathways of CeO_2_/DODAB nanovector.

The uptake efficiency of the nanovector-DNA complexes and intracellular trafficking were evaluated by complexation of Cy3-labeled DNA with CeO_2_/DODAB and by flow cytometry and confocal laser scanning microscopic analyses. Flow cytometric analysis (Fig. 4a,b) showed that around 83.2% of the cells displayed a Cy3-derived red fluorescence signal when CeO_2_/DODAB was used as the vector (mass ratio = 30, N/P = 1). On the other hand, around 48.9% of the cells displayed a Cy3-derived red fluorescence signal (Fig. 4a,b) when only DODAB was used as the vector (N/P = 8). Confocal microscopy analyses were performed to check the intracellular distribution of the CeO_2_/DODAB-Cy3-DNA complexes after different periods. LysoTracker Green was used for labeling the acidic organelles, such as endosomes and lysosomes, in the live cells. Figure 4c shows that after 3 hrs of incubation, the CeO_2_/DODAB-Cy3-DNA complexes successfully entered the cells and co-localized (yellow regions) within the endosomes/lysosomes. The red and green regions consistently overlapped with no separate red regions, indicating that the nanovector/DNA complexes were mostly internalized via the endocytosis pathway. Subsequent escape from the endosomes into the cytoplasm is also crucial for the gene delivery vectors in order to improve the gene delivery efficiency. Interestingly, after 6 hrs of incubation, many separate green and red regions along with yellow regions were apparent, indicating the release of these DNA complexes from the endosomes (Fig. 4d). Both cytosolic and nuclear distribution of Cy3-DNA were observed after transfection with the CeO_2_/DODAB vectors for 6 hrs (Fig. 4d).

To further investigate the cellular uptake pathway mechanism of the CeO_2_/DODAB-pDNA complexes into the HEK293 cells, the cells were pretreated with several endocytosis inhibitors, such as 10 mM NaN_3_ (receptor-mediated endocytosis inhibitor), 0.45 M sucrose (clathrin-mediated endocytosis inhibitor), 10 mM methyl-β–cyclodextrin (MBCD, caveolae-mediated endocytosis inhibitor), and 65 μM LY294002 (macropinocytosis inhibitor). The cells were then transfected with CeO_2_/DODAB-pGL3 complexes (CeO_2_/DODAB to pGL3 mass ratio = 30) in the presence of the added inhibitors for 6 hrs. Pretreatment with NaN_3_, sucrose, and MBCD significantly decreased the Luciferase activities to 84%, 98%, and 92%, respectively, compared to the non-treated cells (Fig. 4e). On the other hand LY294002 pretreatment did not reduce the Luciferase activities (Fig. 4e). Taken together, these results further confirm that the CeO_2_/DODAB-pDNA complexes were internalized through both clathrin- and caveolae-mediated endocytosis.

### *In vivo* transfection efficacy

**Figure 5:**
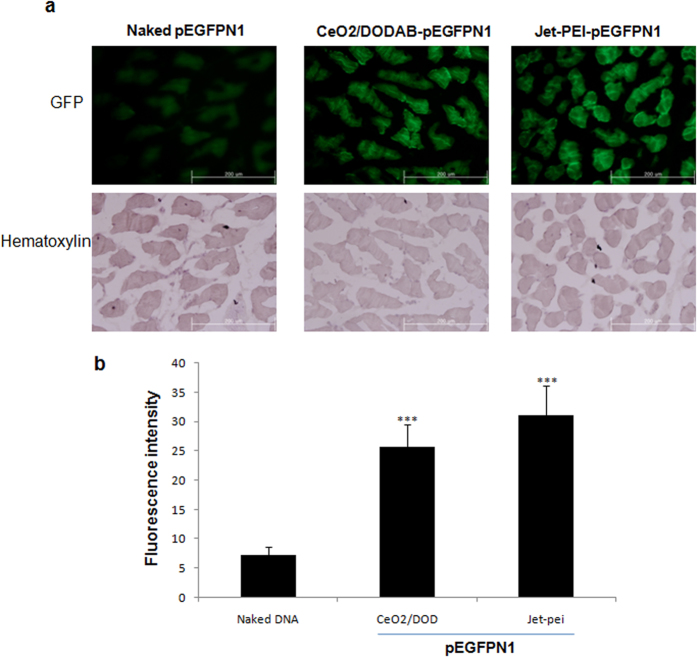
*In vivo* gene transfection. (**a**) EGFP expression in mouse muscles 3 days after intramuscular injections; (**b**) EGFP expression level analyzed by quantifying the fluorescence using the Image J software. All values are expressed as mean ± SD. ***p < 0.001 versus the only pEGFP-treated control group.

pEGFPN1 complexed with CeO_2_/DODAB or *in vivo*-jeiPEI reagents or naked DNA alone were injected into the posterior tibialis muscles of six-week-old male ICR mice. After 72 hrs, the green fluorescence was observed in the muscle sections and the fluorescence intensity was analyzed by Image J software. The fluorescence intensity of muscles transfected with CeO_2_/DODAB and *in vivo*-jeiPEI was 3.5 and 4.3 times higher than the naked DNA treated groups (Fig. 5a,b). However, the *in vivo* transfection efficiency of CeO_2_/DODAB was around 17% less than the commercial *in vivo*-jeiPEI reagents (Fig. 5a,b).

### *In vivo* biocompatibility of CeO_2_/DODAB

CeO_2_/DODAB nanoparticles were injected via tail vein at a dose of 20 mg/kg body weight of ICR male mouse and animal weight was monitored after particle injections. Animals were sacrificed 7 days later and biochemical analysis of the serum was performed. Biochemical analysis of the serum did not show any significant changes in liver function, kidney function, cholesterol and triglycerides of CeO_2_/DODAB versus the saline control groups (Table 2).

## Discussion

In the present study, we prepared a new class of nanoceria (CeO_2_)-based gene delivery vectors, and DODAB (a cationic lipid) was utilized for surface modification of negatively charged CeO_2_ nanoparticles via simple electrostatic interaction. Nanoceria (CeO_2_) was prepared according to the method of Tsai^39^ by simply refluxing ammonium cerium (IV) nitrate and urea. After confirming the synthesis of CeO_2_ by EDS, UV and FTIR spectroscopy, CeO_2_/DODAB was prepared by mixing CeO_2_ and DODAB in a 1:2 mole ratio. Approximately 6.30% of DODAB was bound to the nanoceria surface in CeO_2_/DODAB nanohybrids.

The processes of DODAB deposition on the CeO_2_ surface and complex formation between CeO_2_/DODAB and pDNA were also monitored by UV spectrum, dynamic light scattering (DLS) and zeta potential analyses. CeO_2_/DODAB displayed a red shift (20 nm) in the UV absorption band compared to CeO_2_, which indicated a larger particle size for the latter sample. Similarly, CeO_2_/DODAB-pDNA complexes showed a broad band spanning 315–320 nm with a red shift (5 nm) compared to CeO_2_/DODAB, indicating an even larger particle size than that of CeO_2_/DODAB. The hydrodynamic diameter of CeO_2_ increased by approximately 80 nm when loaded with DODAB, whereas the size of CeO_2_/DODAB increased by approximately 91 nm when complexed with pDNA. The reversal of the zeta potential of CeO_2_ (−24 ± 0.9 mV) relative to that of CeO_2_/DODAB (+41 ± 0.44 mV) indicated the successful deposition of DODAB on the CeO_2_ surface. However, the zeta potential decreased slightly for the CeO_2_/DODAB-pDNA (+36 ± 0.4 mV) complex due to the association with negatively charged DNA. Smaller hydrodynamic size of the CeO_2_/DODAB-pDNA complexes than those of DODAB-pDNA complexes are essential for their successful cellular internalization and subsequent gene delivery.

A major factor for achieving efficient gene delivery is the extent of binding of DNA to the transfection agent. Cationic lipids reportedly form complexes with DNA via electrostatic interactions^42^. In our present study, CeO_2_/DODAB nanohybrids could successfully bind with pDNA via electrostatic interactions and were able to retard pDNA during agarose gel electrophoresis at mass ratios of 10 or above because of positively charged CeO_2_/DODAB-pDNA complex formation. The tight complexing between DODAB and pDNA on the nanoparticle surface also protected pDNA from enzymatic degradation against nucleases.

The gene transfection efficiency of the CeO_2_/DODAB nanovectors was first checked and compared with DODAB alone in HEK293 cells by Luc gene expression, which is a very sensitive technique for checking the expression of exogenous genes after transfection^43^. The transfection efficiency of CeO_2_/DODAB was 5.5 times higher than that of DODAB as evident from Luc expression analysis at their respective optimal ratios. EGFP gene expression analysis also demonstrated that the transfection efficiency of CeO_2_/DODAB was 1.58 times higher than that of DODAB at their respective optimal ratios.

The biocompatibility of the gene delivery vector is another important issue for gene therapy study. The synthesized CeO_2_/DODAB vectors were found to be nontoxic at a concentration upto 140 μg/mL (which is 2.3 times greater than the concentration at which CeO_2_/DODAB shows optimum transfection efficiency), where as DODAB showed concentration-dependent cytotoxicity from 10–100 μm in HEK293 cells. Several researchers have shown that cationic lipid-mediated cytotoxicity was mainly caused by the inhibition of protein kinase C activity by cationic amphiphiles after incorporation into the plasma membrane^44^,^45^. This amphiphile incorporation might lead to transmembrane pores formation and the resultant disruptions of signal transduction. However, after coating onto nanoparticles, DODAB molecules were fixed onto the surface of CeO_2_ core which may prevent amphiphiles from incorporating into the plasma membrane and avoid the formation of transmembrane pores. Besides, CeO_2_/DODAB did not significantly induce ROS formation as well as any stress responsive and pro-survival signaling pathways after 24 hrs of transfection using 4.7 kb pDsRed-Monomer-N1vector. Therefore, the lower cytotoxicity/greater biocompatibility of the CeO_2_/DODAB vectors compared to DODAB are advantageous for use of the former in safe gene therapy study.

The transfection efficiency of CeO_2_/DODAB nanovector has also been compared with Lipofectamine 2000, DOTAP, DEAE-Dextran and calcium phosphate transfection methods in HEK293 cells. The transfection efficiency of CeO_2_/DODAB was found to be significantly higher than DOTAP and DEAE-Dextran, but less than that of Lipofectamine 2000 and calcium phosphate. We have also assessed the cellular metabolic activity as an indicator of cell health to evaluate toxicity that might arise from nanoparticles and other transfection reagents during transfection, and we found that except CeO_2_/DODAB, all other transfection reagents significantly decreased the cell viability compared to untreated control in HEK293 cells. The overall vector performance, also known as Transfection Index (TI) was checked by calculating the product of transfection efficiency and cell viability (Figueroa *et al*.)^46^. This TI will be larger for more biocompatible and efficient vectors, and thus, it was used to determine the overall vector efficiency that combines enhanced transfection with low cytotoxicity^46^. Our results demonstrated that the TI of CeO_2_/DODAB (60%) was comparable with lipofectamine 2000 (56%), and significantly higher than the other transfection reagents in HEK293 cells.

The gene transfection efficiency of the CeO_2_/DODAB nanovectors was also checked by analyzing EGFP gene expression in another two difficult to transfect cell lines, such as MCF-7 and HepG2. The transfection efficiency of CeO_2_/DODAB was found to be significantly higher than calcium phosphate and DEAE-Dextran, but less than that of Lipofectamine 2000 and DOTAP in MCF-7 cells. However, the TI of CeO_2_/DODAB was comparable with Lipofectamine 2000 and DOTAP because of its biocompatible nature, where as Lipofectamine 2000 and DOTAP showed some decrease in cell viability. On the other hand, in HepG2 cells, the transfection efficiency of both CeO_2_/DODAB and Lipofectamine 2000 was very low (around 3%) and all the transfection reagents showed moderate cytotoxic effects.

After that we have also checked whether CeO_2_/DODAB could transfect large plasmid having size more than 10 kb (pTRIPZ, 13.34 kb) in all the above mentioned cell lines. However, the transfection efficiency of CeO_2_/DODAB was very low (12.63%) compared to calcium phosphate (30.4%) and Lipofectamine (60.4%) in HEK293 cells. Besides, the transfection ability of all the above mentioned transfection reagents including CeO_2_/DODAB and Lipofectamine 2000 to transfect a 13.34 kb plasmid was very poor (<1%) in both MCF-7 and HepG2 cells.

All these observations strongly indicate the effectiveness of the CeO_2_/DODAB nanovectors as a gene delivery agent (except in HepG2 cells) to transfect small plasmid (around 5 kb) and its overall transfection performance is comparable with Lipofectamine 2000 and DOTAP, and higher than calcium phosphate and DEAE-dextran.

The increased transfection efficiency of the nanovectors was further supported by the higher cellular uptake efficiency of the nanovector-Cy3-DNA complexes compared with the DODAB-Cy3-DNA complexes. The cellular internalization pathways of the nanovector-DNA complexes were evaluated by intracellular trafficking of nanovector-Cy3-DNA complexes. Confocal laser scanning microscopy analysis illustrated both cytosolic and nuclear distribution of Cy3-DNA after transfection with CeO_2_/DODAB vectors for 6 hrs, indicating successful escape of the nanovector-DNA complexes from the endosomes after cellular internalization.

The higher cellular uptake of nanovector-DNA complexes compared to DODAB-DNA complexes is most possibly due to smaller hydrodynamic size of the former complex. Although the hydrodynamic size of DODAB bilayer is less than 200 nm (data not shown), but form multilamellar structure when complexed with pDNA^47^, and the size become larger than the CeO_2_/DODAB-DNA complexes. After coating onto nanoparticles, DODAB molecules were fixed onto the surface of CeO_2_ core which may prevent them to form large multilamellar structure with DNA, and help to form smaller particles, resulting in enhanced cellular uptake and gene delivery. Besides, the higher cytotoxicity of DODAB compared to CeO_2_/DODAB vectors may also retard the subsequent transcription of the incorporated DNA into mRNA and translation into proteins. The endosomal escape of the CeO2/DODAB-DNA complexes might be due to the interaction of the CeO_2_/DODAB-DNA complexes with the anionic lipid components of the endosome which might disrupt the endosome and thus lead to the escape of DNA^42^.

The cellular internalization pathways of allogeneic materials depend on the cell type and the nature of the transfection agent^48^. The possible cellular uptake pathways of allogeneic materials comprise phagocytosis, endocytosis (clathrin-dependent, caveolae-dependent, or clathrin-/caveolae-independent endocytosis), and macropinocytosis^49^. To investigate the cellular uptake pathways of the CeO_2_/DODAB-pDNA complexes into the HEK293 cells, the cells were pretreated with several endocytosis inhibitors. Because the activity of the inhibitors depends on the administered concentration, we selected effective non-toxic concentrations of the endocytosis inhibitors from previously published articles^42^,^50^,^51^. Our results demonstrated that the nanovector-DNA complexes were internalized through both clathrin- and caveolae-mediated endocytosis.

Finally we have checked the *in vivo* gene transfection efficiency and biocompatibility of the above nanovectors in mice because the ultimate purpose of studying gene delivery is to be used in clinical applications. For *in vivo* gene transfection experiments, we injected naked pEGFPN1 or complexed with either CeO_2_/DODAB or *in vivo*-jeiPEI reagents into the posterior tibialis muscles of ICR mice and the green fluorescence was observed in the muscle sections 3 days later. Quantitative research showed that compared to naked DNA injection, the GFP fluorescence was around 3.5 fold higher for CeO_2_/DODAB, however, the *in vivo* transfection efficiency of CeO_2_/DODAB was only 1.23 fold less than the commercial *in vivo*-jeiPEI reagents.

For *in vivo* safety assessment, CeO_2_/DODAB nanoparticles were injected via tail vein at a dose of 20 mg/kg body weight of ICR mouse and biochemical analysis of the serum was performed 7 days later. It is encouraging that our *in vivo* safety study did not show any signs of toxicity after intravenous injection of CeO2/DODAB nanovectors. Based on its higher biocompatibility and enhanced gene transfection efficiency both *in vitro* and *in vivo*, CeO_2_/DODAB nanohybrids could be considered as a new class of non-viral vectors for therapeutic gene delivery applications.

## Methods

### Materials

Dimethyldioctadecylammonium bromide (DODAB), ammonium cerium(IV) nitrate, urea, calcium chloride, DEAE-dextran hydrochloride and fetal bovine serum (FBS) were purchased from Sigma–Aldrich (St. Louis, MO, USA). Penicillin-streptomycin solution, trypsin-EDTA solution, DMEM, and 1% antibiotic-antimycotic solution were obtained from Life Technologies GIBCO (Grand Island, NY, USA). LysoTracker Green DND-L7526 and lipofectamine 2000 were acquired from Invitrogen, USA. DOTAP and *in vivo*-jetPEI were purchased from Roche Diagnostics, Germany and polyplus transfection, France respectively. Cyanine 3 (Cy3)-labeled 876 bp DNA fragments (a partial sequence of puromycin-resistant gene) was prepared by polymerase chain reaction (PCR) using 5′ Cy3-labeled primers (Macrogen, Korea); GTTTGCGTATTGGGCGCTC, TTAGTCGGGGCTCACTCCTACAG, and pGL4.82 plasmid (Promega, USA) were used as templates. When necessary, highly labeled fragments were obtained by using Cy3-labeled dCTP (PerkinElmer, USA), in place of dCTP, during the PCR reaction. The resultant PCR fragments were purified by 1.0% agarose gel electrophoresis. Plasmid DNA pEGFP-N1 (4.7 kb) and pDsRed-Monomer-N1vector (4.7 kb) were obtained from Clontech (Mountain View, CA, USA). pGL3-Control (5.256 kb) and pTRIPZ (13.34 kb) were obtained from Promega Corp. (Madison, WI, USA) and Open Biosystems (USA), respectively. The Luc assay kit was purchased from Promega (USA). The bicinchoninic acid (BCA) protein assay system was obtained from Thermo Scientific (Rockford, IL, USA). The antibodies used for immunoblotting were against phospho ERK1/2, total ERK1/2, phospho AKT1, total AKT1 (Cell Signaling Technology, Beverly, MA), phospho P38 (Santa Cruz Biotechnology Inc., Santa Cruz, CA), phospho JNK1/2, total JNK1/2 and total P38 (Abcam, Cambridge, MA).

### Preparation of nanoceria (CeO_2_) and surface modification with dimethyldioctadecylammonium bromide (DODAB)

Nanoceria (CeO_2_) was synthesized according to the protocol developed by Tsai^39^. In brief, ammonium cerium(IV) nitrate (2 g) and urea (2 g) were dissolved in 200 g of distilled water and refluxed at 100 °C. The pH of the reaction mixture was maintained at 7.4. Precipitation was initiated after 1 hr and reflux was continued for another 4 hrs. The mixture solution was then cooled and centrifuged to obtain a pale white precipitate. The slurry was washed thrice with water to remove excess reagents and oven dried at 80 °C overnight. A stock solution of nanoceria was prepared by dissolving the appropriate amount of nanoceria in distilled water, followed by sonication; the solution was kept at room temperature. The synthesized nanoceria was characterized by energy-dispersive X-ray spectroscopy (Oxford EDS-6636) and Fourier transform infrared (FTIR) spectroscopy (Perkin Elmer Spectroscopy GX, PerkinElmer Inc., Waltham, MA, USA).

The DODAB solution was prepared by dissolving an appropriate amount of DODAB (2 mol) in water with subsequent sonication at 50 °C to obtain a clear solution. The DODAB solution (2 mole) was then combined with the nanoceria suspension (1 mole, pH 7.4)) and stirred vigorously for 30 min. The mixture solution was subsequently centrifuged at 14000 g for 10 min to remove excess DODAB and the slurry was washed thrice with water. The slurry was resuspended in distilled water and kept at room temperature. The amount of DODAB bound to the nanoceria surface was calculated by elemental analysis (FlashEA 1112 NC analyzer, Thermo Fisher). The detailed calculation process of the amount of DODAB bound to the nanoceria surface is described below:

The molecular weight of DODAB is 630.95 g and one molecule of DODAB contains one nitrogen (N) atom. Therefore 14 g N is present in 630.95 g DODAB. From the Elemental analysis study, we found that approximately 0.14% N was bound to nanoceria. In the CeO_2_/DODAB nanocomplex, the entire nitrogen (N) came from DODAB. Therefore, 14 g N is present in 630.95 g DODAB. So, 0.14 g N is present in = (630.95 × 0.14)/14 g DODAB = 6.3095 gm DODAB. Therefore, in 100 g CeO_2_/DODAB nanocomplex, the amount of DODAB present = 6.3095 g. So, the percentage of DODAB bound to CeO_2_ surface is 6.3095.

### Preparation of nanoparticle-pDNA complexes

The CeO_2_/DODAB-pDNA complexes were prepared with different masses of CeO_2_/DODAB to 1 μg pDNA. Both the nanovectors and pDNA solutions were prepared in 50 μL deionized (DI) water and then combined to give a final volume of 100 μL. The polyplex solutions were then vortexed gently for 10 sec and incubated for 30 min at room temperature.

### UV spectroscopy, transmission electron microscopy (TEM), dynamic light scattering, and zeta potential measurements

The UV-visible spectra of CeO_2_, CeO_2_/DODAB, and the CeO_2_/DODAB-pDNA complexes were acquired by using an Optizen POP (Mecasys, *South Korea) instrument.* The primary size of CeO_2_, CeO_2_/DODAB, and the CeO_2_/DODAB-pDNA complexes was measured by transmission electron microscopy (TEM) using a JEM-1200EX microscope and at an accelerating voltage of 300 kV. The hydrodynamic size and zeta potential of CeO_2_, CeO_2_/DODAB, DODAB/pDNA (N/P = 8) and the CeO_2_/DODAB-pDNA (mass ratio = 30) complexes were measured in water by using a Zetasizer Nano ZS90 (Malvern Instruments, Ltd., UK) instrument. For the CeO_2_/DODAB-pDNA complex, CeO_2_/DODAB to pDNA mass ratio of 30 was used.

### Agarose gel retardation and DNase I protection assay

The pDNA binding ability of the nanovectors was determined by agarose gel (1.0% w/v) retardation assay. The agarose gel was prepared in Tris-acetate-EDTA buffer containing ethidium bromide (0.1 mg/mL). The CeO_2_/DODAB-pDNA complexes were prepared with various mass ratios using 100 ng pDNA. Gel electrophoresis was carried out at 80 V and the location of pDNA in the gel was analyzed using a UV transilluminator (UVP, Bio Doc-It). To evaluate the stability of the CeO_2_/DODAB-pDNA complexes against nucleases, the complex solutions were incubated with 1 μL DNase I (2 U/μL) in 50 mM Tris-Cl and 10 mM MgCl_2_ at pH 7.4 at a temperature of 37 °C over the course of 30 min. The DNase I was then inactivated by adding 1 μL of 100 mM ethylenediaminetetraacetic acid (EDTA). The integrity of pDNA was analyzed by 1% agarose gel electrophoresis. For quantitative analysis, the pDNA was then released from the CeO_2_/DODAB-pDNA complex after DNase I treatment by adding 1% sodium dodecyl sulfate (SDS) obtained from Thermo Scientific (Rockford, IL, USA). The band intensity of pristine pDNA and the pDNA released from the CeO_2_/DODAB-pDNA complex after DNase I treatment followed by addition of 1% SDS were compared with Image J software.

### Cell culture

Human embryonic kidney (HEK293) cells, MCF-7 human breast cancer and HepG2 human liver cancer cells were cultured in DMEM that was supplemented with 10% FBS and 100 U/mL penicillin-streptomycin; the cells were cultured in a humidified incubator maintained at 37 °C in the presence of 5% CO_2_.

### Gene transfection: Expression of Luc gene in HEK293 cells

HEK293 cells were seeded (5 × 10^4^ cells/well) into 24-well, flat bottom culture plates and incubated overnight at 37 °C in a 5% CO_2_ incubator. The transfection experiments were carried out when the cells reached 50–60% confluence and 1 μg pGL3 was used for each well. Prior to transfection, the medium was exchanged with 400 μL of fresh DMEM without FBS and antibiotics. Subsequently, 100 μL of the CeO_2_/DODAB-pGL3 complex solutions (CeO_2_/DODAB to pGL3 mass ratio = 10–100) or DODAB/pGL3 complex solutions (nitrogen to phosphate ratio = 6–10) was added to the cells and transfected for 6 hrs in a humidified incubator at 37 °C in the presence of 5% CO_2_. The medium was then replaced with fresh medium containing 10% FBS and antibiotics and cultured for 48 hrs. The Luciferase activities were measured using Luciferase assay according to the manufacturer’s (Promega) protocol. The gene delivery efficiency is herein expressed in relative light units per gram of total protein (RLU/g) from each sample.

### Expression of enhanced green fluorescent protein (EGFP) gene in HEK293, MCF-7 and HepG2 cells

The gene transfection efficacy of nanoceria was also checked by EGFP expression analysis and compared with other transfection reagents, such as Lipofectamine 2000, DOTAP, DEAE-Dextran and calcium phosphate. The HEK293 cells were seeded and transfected as described above, with the exception that pEGFP-N1 was used instead of pGL3. Cells were transfected with the CeO_2_/DODAB-pEGFP-N1 complexes (CeO_2_/DODAB to pEGFP-N1 mass ratio = 10–60) or DODAB/pEGFP-N1 complex solutions (nitrogen to phosphate ratio = 8) for 6 hrs. For the comparison of transfection efficiency of CeO_2_/DODAB and other transfection reagents, such as Lipofectamine 2000, DOTAP, DEAE-Dextran and calcium phosphate, HEK293, MCF-7 and HepG2 cells were seeded as above and transfected with CeO_2_/DODAB (30 μg), lipofectamine 2000 (5 μL), DOTAP (10 μL), DEAE-dextran (final concentration, 40 μg/mL) and calcium phosphate (DNA suspended in 250 mM CaCl_2_ and mixed with equal volume of 2X hepes buffered saline) using 1 μg pEGFP-N1 for 6 hrs. Forty-eight hours after transfection, the cells were observed by using an inverted fluorescence microscope. The efficiency of gene delivery was also quantified by flow cytometry using FACS Calibur and the data were analyzed with Cell Quest software.

### Cytotoxicity assay

Cells were seeded (1.5 × 10^4^ cells/well) into 96-well, flat-bottom culture plates and incubated for 24 hrs at 37 °C in a 5% CO_2_ incubator. The used medium was replaced with fresh DMEM containing no FBS and antibiotics. The cells were then treated with CeO_2_/DODAB (40–200 μg/mL), DODAB (10–100 μM), lipofectamine 2000 (10 μL/mL), DOTAP (20 μL/mL), DEAE-dextran (40 μg/mL) and calcium phosphate (100 μL 250 mM CaCl_2_ mixed with 100 μL 2X hepes buffered saline and volume made up to 1 mL by the addition of DMEM media) for 6 hrs in a humidified incubator at 37 °C in the presence of 5% CO_2_. The medium was then replaced with fresh medium containing 10% FBS and antibiotics. A cell viability assay was performed using the Cell Counting Kit-8 (CCK-8, Dojindo Laboratories, Kumamoto, Japan) after 48 hrs. The absorbance was read at a wavelength of 450 nm using a microtiter plate reader (Multiskan FC, *Thermo Fisher Scientific Inc*., Waltham, MA, USA).

### Reactive oxygen species (ROS) formation analysis

HEK293 cells were first transfected with 4.7 kb pDsRed-Monomer-N1vector (obtained from Clontech, Mountain View, CA, USA) using CeO_2_/DODAB. After 24 hrs of transfection, cells were incubated with 10 μM 2′,7′-dichlorodihydrofluoresceindiacetate (H_2_-DCFDA) (Sigma-Aldrich, St. Louis, MO) in a humidified incubator at 37 °C for 30 min, washed with phosphate-buffered saline (PBS) and resuspended in PBS. The number of DCF-positive cells was quantified by flow cytometry using FACS Calibur and the data were analyzed with Cell Quest software.

### Immunoblotting

HEK293 cells were first transfected with 4.7 kb pDsRed-Monomer-N1vector (obtained from Clontech, Mountain View, CA, USA) using CeO_2_/DODAB. After 24 hrs of transfection, cells were lysed in radioimmunoprecipitation (RIPA) lysis buffer containing protease and phosphatase inhibitors. Equal amounts of protein were resolved by 12% sodium dodecyl sulfate-polyacrylamide gel electrophoresis (SDS-PAGE) and proteins were electrophoretically transferred to PVDF membranes. Membranes were blocked at room temperature with 6% non-fat dry milk for 2 hrs to prevent non-specific binding, and then incubated with primary antibodies overnight at 4 °C. Immunoreactivity was detected through sequential incubation with horseradish peroxidase-conjugated secondary antibodies and enhanced chemiluminescence reagents.

### Expression of red fluorescent protein (RFP) gene in HEK293, MCF-7 and HepG2 cells

The HEK293, MCF-7 and HepG2 cells were seeded and transfected as described above using 1 μg of 13.34 kb pTRIPZ for 6 hrs. After transfection, the medium was replaced with fresh medium containing 10% FBS, antibiotics and 1 μg/mL doxycycline and cultured for 48 hrs. Forty-eight hours after transfection, the cells were observed by using an inverted fluorescence microscope. The efficiency of gene delivery was also quantified by flow cytometry using FACS Calibur and the data were analyzed with Cell Quest software.

### Cellular uptake and distribution of CeO_2_/DODAB-Cy3-labeled DNA complexes in HEK293 cells

To check the intracellular uptake capacity of the nanovector-DNA complexes, Cy3-labeled DNA (1 μg/well) was complexed with CeO_2_/DODAB (CeO_2_/DODAB to DNA mass ratio = 20–40) or DODAB (nitrogen to phosphate ratio = 8) and added to the HEK293 cells in FBS and antibiotic-free DMEM medium and then incubated for 6 hrs. The cells were seeded into 24-well culture plates as described earlier. The cells were then washed thrice with PBS and trypsinized. The cells were finally resuspended in PBS and the intracellular uptake capacity was examined by flow cytometry (FACS Calibur); the data were analyzed with Cell Quest software.

To evaluate the intracellular distribution of the nanovector-DNA complexes, the cells were incubated with CeO_2_/DODAB-Cy3-labeled DNA complexes (CeO_2_/DODAB to DNA mass ratio = 30) for 3 or 6 hrs. The cells were then incubated with LysoTracker Green for 30 min and washed thrice with PBS to eliminate the background signals. The cells were observed by confocal laser scanning microscopy.

### Cellular uptake pathways in HEK293 cells

The HEK293 cells were seeded into 24-well culture plates as described earlier. When the cells reached 60–70% confluence, they were pretreated with several endocytosis inhibitors, such as 10 mM NaN_3_ (Sigma–Aldrich, St. Louis, MO, USA), 0.45 M sucrose (Junsei Chemical Co., Ltd., Japan), and 10 mM methyl-β-cyclodextrin (Sigma–Aldrich, St. Louis, MO, USA), as well as a macropinocytosis inhibitor, such as 65 μM LY294002 (Sigma–Aldrich, St. Louis, MO, USA) for 30 min^42^,^50^,^51^. The CeO_2_/DODAB-pGL3 complexes (CeO_2_/DODAB to pGL3 mass ratio = 30) were then added to the cells and transfected for 6 hrs as described earlier. The Luciferase activities were measured after 48 hrs using Luciferase assay according to the manufacturer’s (Promega) protocol.

### *In vivo* transfection

ICR male mice were housed in wire cages at 22 ± 1 °C with 70% humidity under a 12/12 hrs light–dark cycle. Animals had access to food and water ad libitum. This study was carried out in strict accordance with the recommendations in the Guide for the Care and Use of the Konkuk University Animal Care and Experimentation Community. All experimental protocols were approved by the Committee on the Ethics of Animal Experiments of the Konkuk University (IACUC approval number: KU11035). Five microgram of pEGFPN1 complexed with CeO_2_/DODAB or *in vivo*-jeiPEI reagents or naked DNA alone was injected into the posterior tibialis muscles of six-week-old male ICR mice. After three days, the muscles were isolated and embedded with Optimal Cutting Temperature (OCT) Compound (Sakura Finetek USA) and froze at −80 °C. Frozen sections of 5 μm thick were cut and stained with hematoxylin. The fluorescence of EGFP was observed by using an inverted fluorescence microscope and the fluorescence intensity was analyzed by Image J software.

### *In vivo* CeO_2_/DODAB toxicity testing

The ICR male mice (six-week-old) were randomly divided into two groups: CeO_2_/DODAB and the saline control group with three mice per group. We used 20 mg/kg nanoparticle for intravenous injection through tail vein and animal weight was monitored after particle injections. Animals were sacrificed 7 days later to obtain blood. The serum was obtained by centrifuging the whole blood at 3000 rpm for 15 min and biochemical parameters were assayed.

### Statistical analysis

All experiments were performed at least in triplicate, and statistical analyses were performed by one-way analysis of variance (ANOVA) followed by a Student’s *t*-test. The level of significance was set at *p < 0.05, **p < 0.01, and ***p < 0.001.

## Additional Information

**How to cite this article**: Das, J. *et al*. Cationic lipid-nanoceria hybrids, a novel nonviral vector-mediated gene delivery into mammalian cells: investigation of the cellular uptake mechanism. *Sci. Rep.* **6**, 29197; doi: 10.1038/srep29197 (2016).

## Supplementary Material

Supporting Information

## Figures and Tables

**Table 1:** Hydrodynamic diameter and surface zeta potential of CeO_2_, CeO_2_/DODAB and CeO_2_/DODAB-pDNA complexes.

Vectors/Complexes	Zeta potential (mV)	Diameter (nm)
CeO_2_	−24 ± 0.9	291 ± 7
CeO_2_/DODAB	+41 ± 0.44	370 ± 19
CeO_2_/DODAB pEGFPN1	+36 ± 0.4	461 ± 14
CeO_2_/DODAB-pGL3	+34 ± 0.4	464 ± 74
DODAB-pGL3	+37 ± 0.6	583 ± 72

**Table 2:** *In vivo* biocompatibility of nanoceria/DODAB in mice.

NAME	Albumin (g/dL)	T.Bilirubin (mg/dL)	ALP (U/L)	Cholesterol (mg/dL)	Triglyceride (mg/dL)	BUN (mg/dL)	Creatinine mg/dL)
Cont	2.87 ± 0.21	<0.1	35.67 ± 18.72	119.33 ± 17.5	45.67 ± 21.22	19.10 ± 1.54	0.13 ± 0.03
CeO_2_/DODAB	2.97 ± 0.15	<0.1	27.33 ± 7.5	121.67 ± 8.33	49.33 ± 15.3	21.37 ± 2.77	0.14 ± 0.02

All values are expressed as mean ± SEM. (*n* = 3).

## References

[b1] Sorgi F. L., Bhattacharya S. & Huang L. Protamine sulfate enhances lipid-mediated gene transfer. Gene Ther 4, 961–968 (1997). 9349433 10.1038/sj.gt.3300484

[b2] Conner S. D. & Schmid S. L. Regulated portals of entry into the cell. Nature 422, 37–44 (2003). 12621426 10.1038/nature01451

[b3] Hu W. W., Wang Z., Hollister S. J. & Krebsbach P. H. Localized viral vector delivery to enhance *in situ* regenerative gene therapy. Gene Ther 14, 891–901 (2007). 17344901 10.1038/sj.gt.3302940

[b4] Kay M. A., Glorioso J. C. & Naldini L. Viral vectors for gene therapy: the art of turning infectious agents into vehicles of therapeutics. Nat Med 7, 33–40 (2001) 11135613 10.1038/83324

[b5] Waehler R., Russell S. J. & Curiel D. T. Engineering targeted viral vectors for gene therapy. Nat Rev Genet 8, 573–587 (2007). 17607305 10.1038/nrg2141 PMC7097627

[b6] Mochizuki S. . The role of the helper lipid dioleoylphosphatidylethanolamine (DOPE) for DNA transfection cooperating with a cationic lipid bearing ethylenediamine. Biochim Biophys Acta 1828, 412–418 (2013). 23092705 10.1016/j.bbamem.2012.10.017

[b7] Rejman J., Oberle V., Zuhorn I. S. & Hoekstra D. Size-dependent internalization of particles via the pathways of clathrin- and caveolae-mediated endocytosis. Biochem J 377, 159–169 (2004). 14505488 10.1042/BJ20031253 PMC1223843

[b8] Nishiyama N. . Light-induced gene transfer from packaged DNA enveloped in a dendrimeric photosensitizer. Nat Mater 4, 934–941 (2005). 16299510 10.1038/nmat1524

[b9] Thomas C. E., Ehrhardt A. & Kay M. A. Progress and problems with the use of viral vectors for gene therapy. Nat Rev Genet 4, 346–358 (2003). 12728277 10.1038/nrg1066

[b10] Guillot-Nieckowski M., Eisler S. & Diederich F. Dendritic vectors for gene transfection. New J Chem 31, 1111–1127 (2007).

[b11] Medina-Kauwe L. K., Xie J. & Hamm-Alvarez S. Intracellular trafficking of nonviral vectors. Gene Ther 12, 1734–1751 (2005). 16079885 10.1038/sj.gt.3302592

[b12] Sokolova V. & Epple M. Inorganic nanoparticles as carriers of nucleic acids into cells. Angew Chem Int Ed 46, 2–16 (2007). 10.1002/anie.200703039 18098258

[b13] Kneuer C. . A nonviral DNA delivery system based on surface modified silica-nanoparticles can efficiently transfect cells *in vitro*. Bioconjug Chem 11, 926–932 (2000). 11087343 10.1021/bc0000637

[b14] Csogor Z., Nacken M., Sameti M., Lehr C. M. & Schmidt H. Modified silica particles for gene delivery. Mat Sci Eng C 23, 93–97 (2003).

[b15] Wisher A. C., Bronstein I. & Chechik V. Thiolated pamam dendrimer-coated CdSe/ZnSe nanoparticles as protein transfection agents. Chem Commun 15, 1637–1639 (2006). 10.1039/b518115a 16583004

[b16] Sandhu K. K., McIntosh C. M., Simard J. M., Smith S. W. & Rotello V. M. Gold nanoparticle- mediated transfection of mammalian cells. Bioconjug Chem 13, 3–6 (2002). 11792172 10.1021/bc015545c

[b17] Thomas M. & Klibanov A. M. Conjugation to gold nanoparticles enhances polyethylenimine’s transfer of plasmid DNA into mammalian cells. Proc Natl Acad Sci USA 100, 9138–9143 (2003). 12886020 10.1073/pnas.1233634100 PMC170885

[b18] Tsai C. Y. . A biological strategy for fabrication of Au/EGFP nanoparticle conjugates retaining bioactivity. Nano Lett 4, 1209–1212 (2004).

[b19] Singh R. . Binding and condensation of plasmid DNA onto functionalized carbon nanotubes: toward the construction of nanotube-based gene delivery vectors. J Am Chem Soc 127, 4388–4396 (2005). 15783221 10.1021/ja0441561

[b20] Liu Y. . Polyethyleniminegrafted multiwalled carbon nanotubes for secure noncovalent immobilization and efficient delivery of DNA. Angew Chem Int Ed 44, 4782–4785 (2005). 10.1002/anie.200500042 15995988

[b21] Kakizawa Y., Miyata K., Furukawa S. & Kataoka K. Size-controlled formation of a calcium phosphate-based organic–inorganic hybrid vector for gene delivery using poly(ethylene glycol)-block-poly(aspartic acid). Adv Mater 16, 699–702 (2004).

[b22] Niu J., Wang K. & Kolattukudy. P. E. Cerium oxide nanoparticles inhibits oxidative stress and NF-κB activation in H9c2 cardiomyocytes exposed to cigarette smoke extract. J Pharmacol Exp Ther 338, 53–61 (2011). 21464334 10.1124/jpet.111.179978 PMC3126650

[b23] Hirst S. M. . Antioxidant and Anti-inflammatory Properties of Cerium Oxide Nanoparticles in J774A.1 murine macrophages. Small 5, 2848–2856 (2009). 19802857 10.1002/smll.200901048

[b24] Schubert D., Dargusch R., Raitano J. & Chan S. W. Neuroprotective role of Cerium oxide nanoparticles in HT22 hippocampal nerve cell line. Biochem Biophys Res Commun 342, 86–91 (2006). 16480682 10.1016/j.bbrc.2006.01.129

[b25] Niu J., Azfer A., Rogers L. M., Wang X. & Kolattukudy P. E. CeO_2_ nanoparticles protect against the progression of cardiac dysfunction and remodeling by attenuation of myocardial oxidative stress, ER stress, and inflammatory processes. Cardiovasc Res 73, 549–559 (2007).17207782

[b26] Amin K. A., Hassan M. S., Awad el-S. T. & Hashem & The protective effects of cerium oxide nanoparticles against hepatic oxidative damage induced by monocrotaline. Int J Nanomedicine 6, 143–149 (2011). 21289991 10.2147/IJN.S15308 PMC3026579

[b27] Colon J. . Cerium oxide nanoparticles protect gastrointestinal epithelium from radiation-induced damage by reduction of reactive oxygen species and upregulation of superoxide dismutase 2. Nanomedicine 6, 698–705 (2010). 20172051 10.1016/j.nano.2010.01.010

[b28] Korsvik C., Patil S., Seal S. & Self W. T. Superoxide dismutase mimetic properties exhibited by vacancy engineered ceria nanoparticles. Chem Commun 14, 1056–1058 (2007). 10.1039/b615134e 17325804

[b29] Heckert E. G., Karakoti A. S., Seal S. & Self W. T. The role of cerium redox state in the SOD mimetic activity of nanoceria. Biomaterials 18, 2705–2709 (2008). 18395249 10.1016/j.biomaterials.2008.03.014 PMC2396488

[b30] Hochella M. F. Jr. . Nanominerals, mineral nanoparticles, and earth systems. Science 319, 1631–1635 (2008). 18356515 10.1126/science.1141134

[b31] Yu J. C., Zhang L. & Lin J. Direct sonochemical preparation of high-surface-area nanoporous ceria and ceria-zirconia solid solutions. J Colloid Interface Sci 260, 240–243 (2003). 12742056 10.1016/s0021-9797(02)00168-6

[b32] Liu B., Sun Z., Huang P. J. & Liu J. Hydrogen peroxide displacing DNA from nanoceria: mechanism and detection of glucose in serum. J Am Chem Soc 137, 1290–1295 (2015). 25574932 10.1021/ja511444e

[b33] Rose J. K., Buonocore L. & Whitt M. A. A new cationic liposome reagent mediating nearly quantitative transfection of animal-cells. Biotechniques 10, 520–525 (1991). 1867862

[b34] You J., Kamihira M. & Iijima S. Surfactant-mediated gene transfer for animal cells. Cytotechnology 25, 45–52 (1997). 22358878 10.1023/A:1007955631313 PMC3466739

[b35] Birchall J. C., Kellaway I. W. & Mills S. N. Physico-chemical characterisation and transfection efficiency of lipid-based gene delivery complexes. Int J Pharm. 183, 195–207 (1999). 10361170 10.1016/s0378-5173(99)00117-9

[b36] You J., Kamihira M. & Iijima S. Enhancement of transfection efficiency by protamine in DDAB lipid vesicle-mediated gene transfer. J Biochem 125, 1160–1167 (1999). 10348920 10.1093/oxfordjournals.jbchem.a022399

[b37] Mizuarai S., Ono K., You J., Kamihira M. & Iijima S. Protamine-modified DDAB lipid vesicles promote gene transfer in the presence of serum. J Biochem 129, 125–132 (2001). 11134966 10.1093/oxfordjournals.jbchem.a002822

[b38] Dass C. R., Walker T. L. & Burton M. A. Liposomes containing cationic dimethyl dioctadecyl ammonium bromide: formulation, quality control, and lipofection efficiency. Drug Deliv 9, 11–18 (2002). 11839204 10.1080/107175402753413136

[b39] Tsai M. S. Powder synthesis of nano grade cerium oxide via homogenous precipitation and its polishing performance. Materials Science and Engineering B 110, 132–134 (2004).

[b40] Lee S. K., Han M. S., Asokan S. & Tung C. H. Effective Gene Silencing by Multilayered siRNA-Coated Gold Nanoparticles. Small 7, 364–370 (2011). 21294265 10.1002/smll.201001314 PMC3099143

[b41] Pecora R. Dynamic Light Scattering Measurement of Nanometer Particles in Liquids. J Nanopart Res 2, 123–131 (2000).

[b42] Li P., Li D., Zhang L., Li G. & Wang E. Cationic lipid bilayer coated gold nanoparticles-mediated transfection of mammalian cells. Biomaterials 29, 3617–3624 (2008). 18571230 10.1016/j.biomaterials.2008.05.020

[b43] Nguyen V. T., Morange M. & Bensaude O. Firefly luciferase luminescence assays using scintillation counters for quantitation in transfected mammalian cells. Anal Biochem 171, 404–408 (1988). 3407940 10.1016/0003-2697(88)90505-2

[b44] Farhood H., Bottega R., Epand R. M. & Huang L. Effect of cationic cholesterol derivatives on gene-transfer and protein-kinase-c activity. Biochim Biophys Acta 1111, 239–246 (1992). 1420259 10.1016/0005-2736(92)90316-e

[b45] Zelphati O. & Szoka F. C. Mechanism of oligonucleotide release from cationic liposomes. Proc Natl Acad Sci USA 93, 11493–11498 (1996). 8876163 10.1073/pnas.93.21.11493 PMC38085

[b46] Figueroa E. R. . Optimization of PAMAM-gold nanoparticle conjugation for gene therapy. Biomaterials 35, 1725–1734 (2014). 24286816 10.1016/j.biomaterials.2013.11.026 PMC3906732

[b47] Silva J. P. . DODAB:monoolein-based lipoplexes as non-viral vectors for transfection of mammalian cells. Biochim Biophys Acta 1808, 2440–2449 (2011). 21787746 10.1016/j.bbamem.2011.07.002

[b48] Conner S. D. & Schmid S. L. Regulated portals of entry into the cell. Nature 422, 37–44 (2003). 12621426 10.1038/nature01451

[b49] Doherty G. J. & McMahon H. T. Mechanisms of endocytosis. Annu Rev Biochem 78, 857–902 (2009). 19317650 10.1146/annurev.biochem.78.081307.110540

[b50] Du B., Tian L., Gu X., Li D. . Anionic Lipid, pH-Sensitive Liposome-Gold Nanoparticle Hybrids for Gene Delivery -Quantitative Research of the Mechanism. Small 11, 2333–2340 (2015). 25594807 10.1002/smll.201402470

[b51] Sokolova V. . Mechanism of the uptake of cationic and anionic calcium phosphate nanoparticles by cells. Acta Biomater 9, 7527–7535 (2013). 23454056 10.1016/j.actbio.2013.02.034

