# Supplementary material for: Cationic lipid-nanoceria hybrids, a novel nonviral vector-mediated gene delivery into mammalian cells: investigation of the cellular uptake mechanism
Source: Sci Rep. 2016 Jul 6;6:29197. doi: 10.1038/srep29197 (PMC4933920; doi:10.1038/srep29197)
Supplement: Supporting Information [file srep29197-s1.doc]

**Cationic lipid-nanoceria hybrids, a novel nonviral vector-mediated gene delivery into mammalian cells: investigation of the cellular uptake mechanism**

Joydeep Das, Jae Woong Han, Yun-Jung Choi, Hyuk Song, Ssang-Goo Cho, Chankyu Park, Han Geuk Seo, and Jin-Hoi Kim*

Dept. of Stem Cell and Regenerative Biology, Humanized Pig Research Center (SRC), Konkuk University, Seoul 143-701, South Korea

**Running title:** Nanoceria mediated gene delivery

**Corresponding author:**

**Jin-Hoi Kim*:** [jhkim541@konkuk.ac.kr](mailto:jhkim541@konkuk.ac.kr)

**Permanent address:** Dept.of Stem Cell and Regenerative Biology, Humanized Pig Research Center (SRC), Konkuk University, Seoul 143-701, South Korea


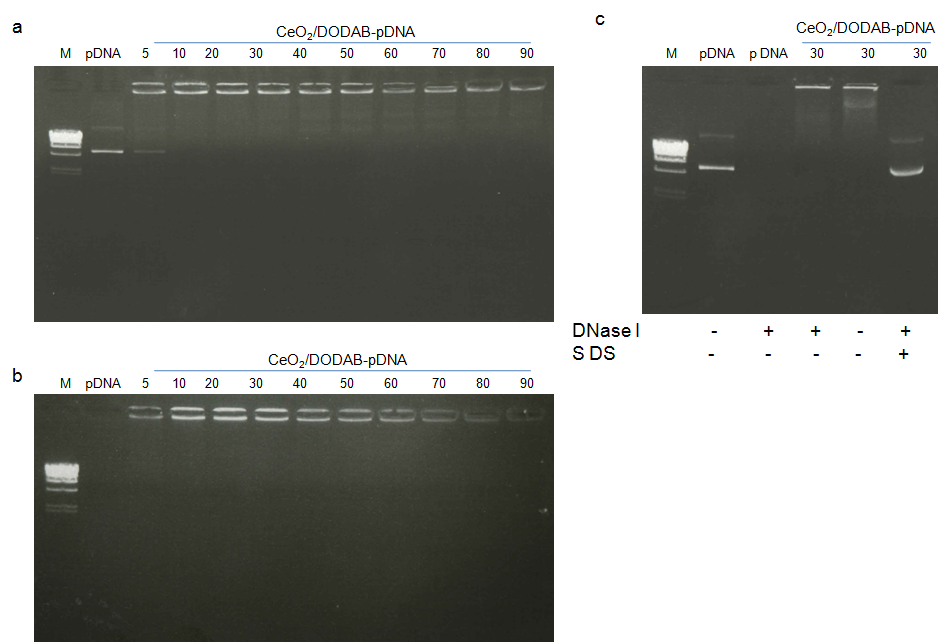


**Supplementary Figure 1.** **Agarose gel retardation assay.** (A) In absence of DNase I. The CeO_2_/DODAB-pDNA complexes were prepared with various mass ratios using 100 ng pDNA; (B) protection of pDNA against DNase I treatment; (C) pDNA release from CeO_2_/DODAB–pDNA complexes (mass ratio = 30) after DNase I digestion followed by SDS treatment. M: DNA marker.


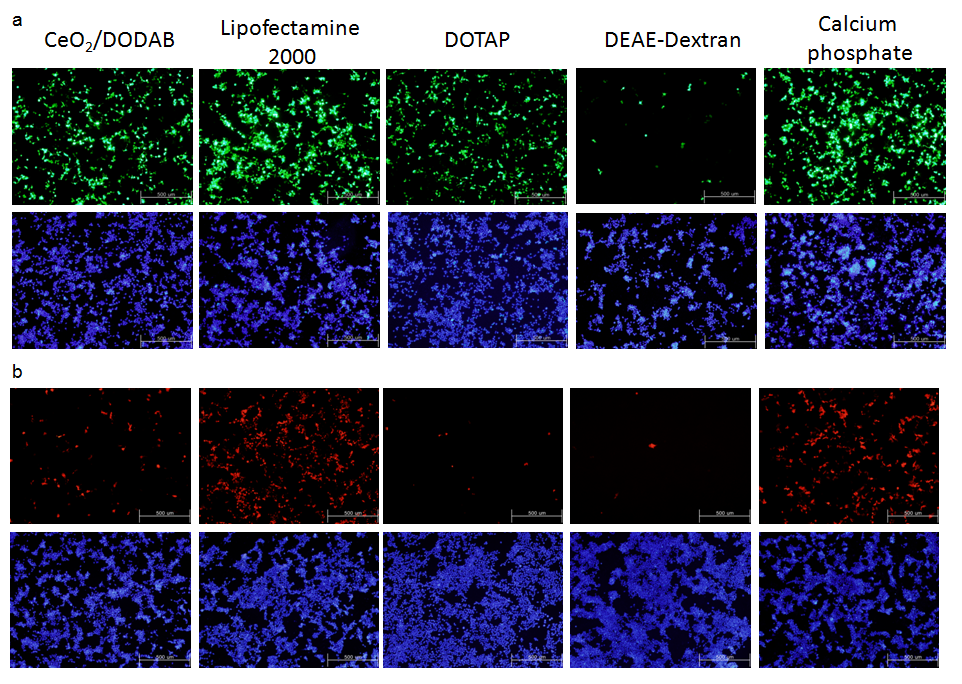


**Supplementary Figure 2. Fluorescence microscopic images after transfection with pEGFP-N1 (4.7 kb) and pTRIPZ (13.34 kb) in HEK293 cells.** (a) Fluorescence microscopic images after transfection with pEGFP-N1 (4.7 kb); (b) Fluorescence microscopic images after transfection with pTRIPZ (13.34 kb).


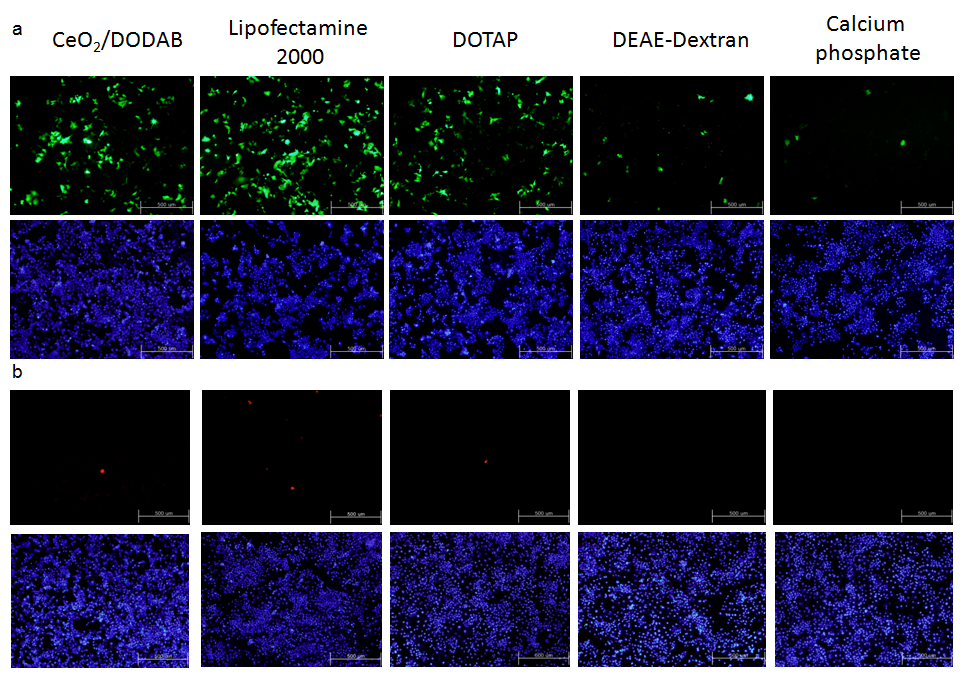


**Supplementary Figure 3. Fluorescence microscopic images after transfection with pEGFP-N1 (4.7 kb) and pTRIPZ (13.34 kb) in MCF-7 cells.** (a) Fluorescence microscopic images after transfection with pEGFP-N1 (4.7 kb); (b) Fluorescence microscopic images after transfection with pTRIPZ (13.34 kb).

**
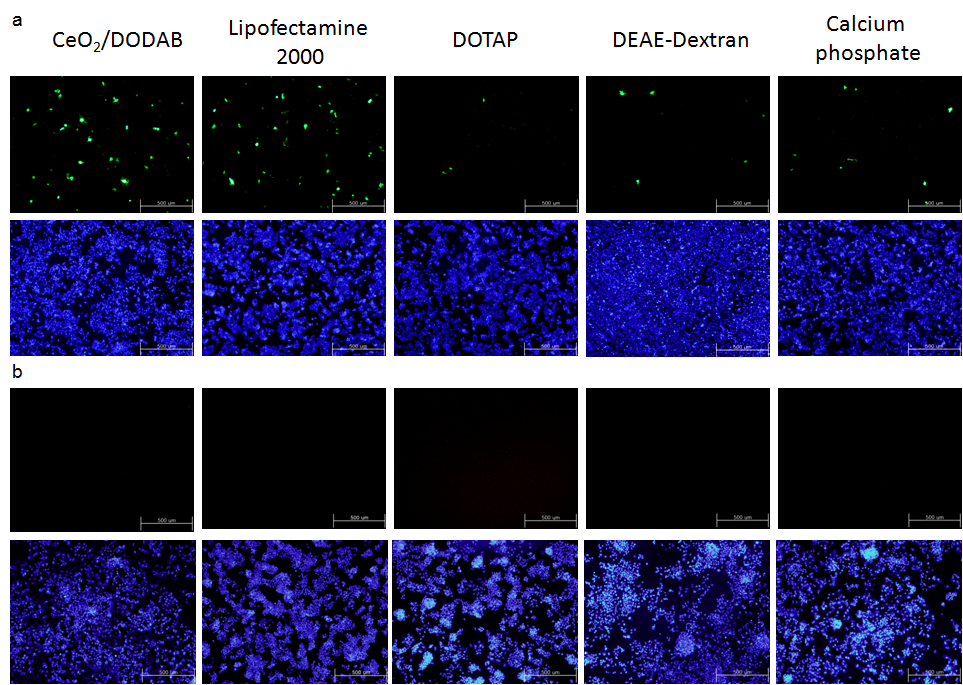
**

**Supplementary Figure 4. Fluorescence microscopic images after transfection with pEGFP-N1 (4.7 kb) and pTRIPZ (13.34 kb) in HepG2 cells.** (a) Fluorescence microscopic images after transfection with pEGFP-N1 (4.7 kb); (b) Fluorescence microscopic images after transfection with pTRIPZ (13.34 kb).

**Supplementary Table 1: Elemental analysis of CeO_2_/DODAB nanovector**

| **Nanovector** | **% of nitrogen** | **% DODAB** |
| --- | --- | --- |
| **CeO_2_/DODAB**  **[(CH_3_)_4_(CH_2_)_34_NBr]** | **0.1398** | **6.30** |

**Supplementary Table 2:** Conversion of mass ratios of CeO_2_/DODAB to pDNA in CeO_2_/DODAB-pDNA complexes into N/P ratios

| **Mass ratio** | **N/P ratio** |
| --- | --- |
| 10 | 0.33 |
| 20 | 0.66 |
| 30 | 1.0 |
| 40 | 1.33 |
| 60 | 2.0 |
| 80 | 2.66 |
| 100 | 3.33 |
